# Clinical outcome after IMSI procedure in an unselected infertile population: a pilot study

**DOI:** 10.1186/1742-4755-10-16

**Published:** 2013-03-22

**Authors:** Roberto Marci, Fabien Murisier, Giuseppe Lo Monte, Ilaria Soave, Alain Chanson, Françoise Urner, Marc Germond

**Affiliations:** 1 Department of Growth and Reproduction, Section of Gynecology and Obstetrics Infertility Unit, Sant'Anna University Hospital, via Aldo Moro 8, 44100, Cona (Ferrara), Italy; 2 CPMA, Centre of Reproductive Medicine, Rue de la Vigie 5, Lausanne 1003, Switzerland; 3 FERTAS Andrology Laboratory, Rue de la Vigie 5, Lausanne 1003, Switzerland; 4 FABER Foundation, Rue de la Vigie 5, Lausanne 1003, Switzerland; 5 Department of Biomedical Sciences and Advanced Therapies, University of Ferrara, Corso Giovecca 183, Ferrara 44121, Italy

**Keywords:** ICSI, IMSI, IVF, Infertility, Sperm morphology, Sperm selection

## Abstract

**Background:**

To date the IMSI procedure represents the only real-time and unstained method available to discard spermatozoa with ultrastructural defects. Several studies demonstrated that IMSI provides positive results in couples with severe male factor infertility or repeated ICSI failures. Aim of this pilot study is to evaluate the differences between IMSI and ICSI in terms of IVF outcomes in an unselected infertile patient population.

**Methods:**

Three hundred and thirty-two couples were analyzed: 281 couples underwent conventional ICSI procedure and 51 underwent IMSI technique.

**Results:**

No statistically significant differences were found between implantation rate (ICSI: 16,83%; IMSI: 16,67%), fertilization rate (ICSI: 77,27%; IMSI: 80,00%) and pregnancy rate (ICSI: 25,30%; IMSI: 23,50%). Both groups were comparable when considering live birth rate (ICSI: 11,39%; IMSI:13,72%), ongoing pregnancy rate (ICSI: 7,47%; IMSI: 5,88%) and miscarriage rate (ICSI: 17,78; IMSI: 5,26%). The subgroup analyses did not show a statistical difference between ICSI and IMSI neither in male factor infertility subgroup nor in patients with more than one previous ICSI attempt. A trend towards better laboratory and clinical outcomes was detected in the male factor infertility subgroup when IMSI was applied.

**Conclusions:**

Our preliminary results show that the IMSI technique does not significantly improve IVF outcomes in an unselected infertile population.

## Background

**Figure 1:**
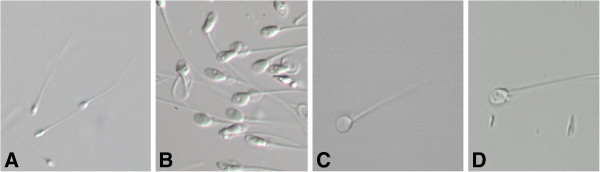
**Live spermatozoa observed under conventional ICSI conditions (A) and IMSI magnification (B, C, D).** Shape and presence of vacuoles can be clearly observed in **B**. A vacuole-free spermatozoon is shown in **C**.

Introcytoplasmic sperm injection (ICSI) represents the recommended treatment in many cases of infertility and is characterized by a pregnancy rate per cycle of about 30% [1]. The power of ICSI as a tool to overcome fertilization failures in IVF procedures has led to a first common acceptance by embryologists that morphology of the selected sperm for injection was of secondary importance [2]. The selection process preceding the micro-injection of a motile, normal-looking spermatozoon into the oocyte’s cytoplasm is usually performed under a low magnification (×200/400) that could be responsible for the underestimation of possible subtle sperm organellar malformations. A spermatozoon classified as “normal” after a morphologic evaluation performed at low magnification could carry relevant ultra-structural defects that could interfere with fertilization process and embryo development. In 2002 Bartoov et al. developed a method of human spermatozoa evaluation called “motile sperm organelle morphology examination” (MSOME) [3]. It permits spermatozoa observation at high magnification (>6000x) compared to the 200-400x observed by conventional ICSI using an inverted microscope equipped with Normarski interference contrast optics. Application of this method to patients undergoing conventional IVF/ICSI has led to the development of the intracytoplasmatic morphologically selected sperm injection (IMSI, Figure 1).

Aim of this pilot study is to evaluate the differences between IMSI and ICSI in terms of IVF outcomes in an unselected infertile patient population.

## Methods

From January 2009 to March 2012, 332 couples were analyzed. Couples were divided into two groups by using a randomized computer-generated list as follows: 281 couples underwent conventional ICSI procedure and 51 underwent IMSI technique. All cycles were performed with fresh ejaculated spermatozoa. Patients who required the use of cryopreserved or surgically retrieved semen were excluded from this study. This work has been carried out in accordance with The Code of Ethics of the World Medical Association (Declaration of Helsinki) for experiments involving humans. The study was approved by our clinical board. All patients in both groups were enrolled after signing a written informed consent.

All women were stimulated using 150–375 IU of r-FSH (Gonal-F®, Merck Serono, Switzerland; Puregon®, MSD, Switzerland) from d-2 of the menstrual cycle, followed by daily dose of GnRH antagonist Ganirelix or Cetrorelix (Ganirelix® 0.25 mg, Orgalutran®, MSD, Switzerland; Cetrotide® 0.25 mg, Merck Serono, Switzerland) starting from d-5 of the ovarian stimulation until the day of hCG injection. In order to evaluate the ovarian response E_2_ was monitored and transvaginal ultrasounds (US) were performed every 2–3 days. In both groups ovulation triggering was achieved by using a subcutaneous injection of 10000 IU of human chorionic gonadotropin (Pregnyl®, MSD, Switzerland) as soon as at least 2 follicles of at least 17 mm were detectable on US scan. Oocyte retrieval was performed by transvaginal aspiration 36 hours after hCG administration. Embryo transfers (ETs) were performed 48 hours after oocyte retrieval using a K Jet catheter, Prince catheter, Prince Medical, France. The luteal phase was supported by 200 × 2 mg/day of micronized vaginal progesterone (Utrogestan®, Vifor SA, Switzerland). Serum hCG level was measured 14 days after ET. Clinical pregnancies were confirmed with US scans 28–35 days after ET by the presence of gestational sac and fetal heart activity.

IMSI requires a high magnification inverted microscope (600-1000x). A high magnification objective lens (60x) in combination with Hoffman’s contrast on a standard injection microscope (Olympus IX71) was used. Using a 1.6x magnification enhancer a total magnification of 960x can be achieved. Glass-bottomed dishes are required for the specimen observation in order to achieve the best optical quality associated with the real-time digital image enhancement (Octax CytoScreen TM system, Medical Technology Vertriebs-GmbH, Germany), the setup provides an excellent image quality with a total on-screen magnification of about 5400x. Analysis and selection of motile spermatozoa were performed according to the MSOME criteria described by Bartoov et al. [3]. Laboratory and clinical outcomes were evaluated in the two groups. Subgroups analyses were then performed according either to the presence of a male infertility factor or to the number of previous ICSI attempts.

Results are presented as mean ± standard deviation. Differences in variables were statistically analyzed with the nonparametric Wilcoxon’s signed rank test, Student’s t test and χ2 test, as appropriate. A P value of less than .05 was considered statistically significant.

## Results

The characteristics of the studied population (Table 1) were similar in both IMSI and ICSI groups when considering women’s age, number of previous ICSI treatment failures, mean number of retrieved and injected oocytes and semen parameters (evaluated in accordance to WHO criteria 2010). The majority of couples had male factor infertility in both ICSI and IMSI groups. Both the mean age of male partners and the percentage of prepared sperm motility were statistically different but neither of these parameters influences the outcome of a microinjection technique, according to the published literature [4-6]. No statistically significant differences were found between implantation rate, fertilization rate and pregnancy rate. Both groups were comparable when considering live birth rate, ongoing pregnancy rate and miscarriage rate. In the IMSI group fertilization rate and live birth rate were slightly higher and the miscarriage rate tended to be lower when compared to the ICSI group, although no statistical significance was found. The subgroup analysis is reported in Table 2. No statistical difference was reported neither in male factor infertility subgroup nor in patients with more than one previous ICSI attempt. A trend towards better laboratory and clinical outcomes was detected in the male factor infertility subgroup when IMSI was applied. When patients with more than one previous ICSI failure were treated with IMSI a significantly higher fertilization rate was achieved (84.10% versus 76.55%). However, in the same subgroup was reported a trend towards lower implantation rate, pregnancy rate and live birth rate as well as higher miscarriage rate. Live birth rate reached the statistical significance in the subgroup of patients treated with IMSI with one or no previous ICSI failures.

**Table 1:** Main characteristics of the patients and clinical-laboratory outcomes in IMSI and ICSI groups

	**ICSI**		**IMSI**		**p-value**
	**Count/medium**	**d.s.**	**Count/medium**	**d.s.**	
N° of cycles	281		51		
Women age at Pickup	34,98	3,19	35,65	2,98	0,15
Men age at Pickup	37,61	5,47	39,51	5,23	0,02
N° of ICSI attempts	1,61	0,88	1,55	0,87	0,68
Causes of infertility (%):					
• Tubal factor	8.5		9.8		
• Ovulatory	5.3		1.9		
• Endometriosis	7.5		7.8		
• Male factor	36.6		39.2		
• Multiple factors	20.9		19.6		
• Unexplained	21		21.6		
N° of injected oocytes	8,12	4,42	8,23	3,78	0,84
Native sperm concentration (million/ml)	23,58	29,34	27,75	32,29	0,39
Native sperm motility rate (%)	26,27	17,94	27,56	16,55	0,62
Prepared sperm concentration (million/ml)	16,12	23,94	21,1	21,46	0,14
Prepared sperm motility (%)	54,43	24,62	60,16	16,95	0,04
2 pronuclei day 1	6,27	3,72	6,59	3,57	0,57
N° of transferred embryos	1,86	0,38	1,87	0,33	0,85
Implantation rate (%)	16,83		16,67		0,97
Fertilization rate (%)	77,27		80,00		0,22
Pregnancy rate (%)	30,96		33,33		0,74
Live birth rate (%)	11,39		13,72		0,23
Ongoing pregnancy rate (%)	7,47		5,88		0,69
Miscarriage rate (%)	17,78		5,26		0,17

**Table 2:** Comparison between IMSI and ICSI groups according to the presence of male factor infertility and the number of previous ICSI attempts

	**No male factor infertility**			**Male factor infertility**			**>1 ICSI attempt**			**≤ 1 ICSI attempt**		
	**ICSI**	**IMSI**	**p-value**	**ICSI**	**IMSI**	**p-value**	**ICSI**	**IMSI**	**p-value**	**ICSI**	**IMSI**	**p-value**
**FR**	80.64%	80.54%	NS	73.03%	79.17%	NS	76.55%	84.10%	**< 0.05**	77.75%	78.12%	NS
**IR**	17.87%	14.29%	NS	16.13%	18.18%	NS	17.35%	6.2%	NS	16.44%	22.41%	NS
**PR**	35.40%	27.78%	NS	28.07%	37.14%	NS	30.57%	15.79%	NS	31.25%	43.75%	NS
**MR**	9.73%	11.11%	NS	1.17%	5.7%	NS	10.7%	5.2%	NS	10.62%	12.5%	NS
**LBR**	11.80%	5.55%	NS	11.11%	20%	NS	19%	10%	NS	9.4%	21.87%	**< 0.05**

FR: Fertilization Rate; IR: Implantation Rate; PR: Pregnancy Rate; MR: Miscarriage Rate; LBR: Live Birth Rate; NS: not significant.

## Discussion and conclusions

Several studies demonstrated a positive correlation between optimal sperm morphology evaluation and ICSI outcomes improvement. De Vos et al. [7] evaluated the impact of individual sperm morphology on ICSI outcome (fertilization, embryo development and implantation rate). This study was performed by using an inverted light microscope and sperm cells were classified as normal or abnormal in accordance to Kruger criteria [8]. The authors demonstrated that the injection of an abnormal spermatozoa was associated to a significantly lower fertilization, implantation and pregnancy rate when compared to ICSI cycles performed by injecting a spermatozoa with apparently normal morphology. The low magnification and resolution of the microscope used for the morphology assessment represented the main limitation of this study. At the present time the ICSI technique is performed under a low magnification (×200/400) that could be responsible for the underestimation of possible subtle sperm organellar alterations. Spermatozoa morphology is the best selection criteria for intracytoplasmatic injection. To date the IMSI procedure represents the only real-time and unstained method available to discard spermatozoa with ultrastructural defects. Indeed, most of the enzymatic or genetic tests currently available cannot be performed on a viable, unfixed, spermatozoa. The detection of large nuclear vacuoles at high magnification could be related to DNA fragmentation and denaturation. It is widely accepted that DNA integrity plays an important role in the fertilization process and in the development and implantation of embryos [9,10]. Furthermore, Vanderzwalmen et al. [11] reported a negative influence of spermatozoa with large nuclear vacuoles in the head on the capability of embryos to develop to the blastocyst stage.

Antinori et al. [12] pointed out the need to increase the efficiency of micro-insemination techniques especially in those countries, such as Italy, where the law limits the number of fertilizable oocytes. Several studies demonstrated that IMSI provides positive results in couples with severe male factor infertility or repeated ICSI failures [3,12,13]. In all these studies the clinical pregnancy rate was noticeably improved in the IMSI group. Moreover, the IMSI group result was associated with a lower abortion rate. Besides, other studies do not show any statistical significant improvement in the clinical outcome. Oliveira et al. [14] report no significant differences between fertilization rate, implantation rate and pregnancy rate when comparing the two procedures, although a trend toward lower miscarriage rate and better ongoing pregnancy rate and live birth rate was found in the IMSI group.

Balaban et al. [15], recently analyzed in a prospective randomized study the clinical differences between IMSI and ICSI procedures in an unselected population. To the best of our knowledge, the study mentioned above is the only one in which the population was not selected neither for the presence of a severe male factor infertility nor for a history of repeated previous failed ICSI attempts. IMSI did not provide any significant improvement in the clinical outcome compared to ICSI. However, trends for higher rates of implantation (28.9% versus 19.5%), clinical pregnancy (54.0% versus 44.4%) and live births (43.7% versus 38.3%) were found in the IMSI group. The second aim of the study was to verify whether better results were achieved when the iMSI procedure was applied to the subgroup of male factor infertility. A significantly higher implantation rate was found in IMSI group (29.6% versus 15.2%). The authors also reported a slight but not significant improvement in live birth rate (36.8% versus 28.2%).

In accordance to Balaban et al., our preliminary results came out to be similar in the two groups, suggesting that IMSI does not significantly improve IVF outcomes in an unselected randomized infertile population. As shown by the subgroup analysis, IMSI did not prove superior to ICSI in terms of laboratory and clinical results, even though a positive trend for better fertilization, implantation, pregnancy and live birth rates was detected in the severe male factor infertility subgroup when IMSI was applied. The data obtained from the analysis of patients with previous ICSI failures are not consistent with the results published in other recent studies [3,12,13]. The reliability of these results is though limited by the small sample size of this pilot study, emphasizing the need for further trials on a larger population. Although further studies in that direction are necessary in order to state possible advantages of the IMSI procedure in couples with severe male factor infertility or with repeated failures after conventional ICSI.

In conclusion, in the last decades ICSI has been widely used for the treatment of infertility, in particular in the case of severe male factor infertility. Moreover, in the light of its high fertilization and pregnancy rates, ICSI has gradually replaced conventional IVF and it is now applied as a first-line therapy by many infertility centers. The innovative IMSI procedure has changed the perception of how a spermatozoon, suitable for injection, looks like. To date, the clinical indications are scarce and derived mainly from the medical history of patients. Our preliminary results show that the IMSI technique does not significantly improve IVF outcomes in an unselected randomized infertile population. In particular, the use of IMSI in unselected patients did not significantly improve fertilization, implantation and pregnancy rates when compared to conventional ICSI. Thus, we do not recommend IMSI for non-male factor, normozoospermic patients or couples addressing to IVF for the first time. Furthermore, our work did not show a clear effectiveness of this method in case of severe male factor infertility or couples with previous ICSI failures.

Considering the limited number of studies on this topic, the absence of an adequate standardization of the method and the lack of worldwide accepted selection criteria, IMSI is far to be considered as a routinely used ART technique.

## Competing interests

The authors have no conflicts of interest to declare.

## Authors’ contributions

RM has substantially contributed to data collection, to the diagnostic process, to the preoperative and operative work up linked to the condition of the patient, to preparation, drafting and revising the final version of the manuscript. He also gave an extremely important intellectual support. FM has substantially contributed to design, preparation, drafting and revising the intellectual content of the final version of the manuscript. AC has substantially contributed to design, preparation, drafting and revising the intellectual content of the final version of the manuscript. FU has substantially contributed to design, preparation, drafting and revising the intellectual content of the final version of the manuscript. GL has substantially contributed to design, to preparation, drafting and revising the final version of the manuscript and gave and significant intellectual support. IS has substantially contributed to design, to preparation, drafting and revising the final version of the manuscript and gave and significant intellectual support. MG has substantially contributed to data collection, to the diagnostic process, to the operative work up linked to the condition of the patient, to preparation, drafting and revising the final version of the manuscript. He also gave an extremely important intellectual support. All authors read and approved the final manuscript.
